# CD36 (SR-B2) as master regulator of cellular fatty acid homeostasis

**DOI:** 10.1097/MOL.0000000000000819

**Published:** 2022-02-03

**Authors:** Jan F.C. Glatz, Miranda Nabben, Joost J.F.P. Luiken

**Affiliations:** a Department of Genetics & Cell Biology, Faculty of Health, Medicine and Life Sciences, Maastricht University; b Department of Clinical Genetics, Maastricht University Medical Center+; c Cardiovascular Research Institute Maastricht (CARIM), Maastricht, The Netherlands

**Keywords:** cardiac function, cardiomyopathy, cellular lipid metabolism, cluster of differentiation 36

## Abstract

Transmembrane glycoprotein cluster of differentiation 36 (CD36) is a scavenger receptor class B protein (SR-B2) that serves various functions in lipid metabolism and signaling, in particular facilitating the cellular uptake of long-chain fatty acids. Recent studies have disclosed CD36 to play a prominent regulatory role in cellular fatty acid metabolism in both health and disease.

**Recent findings:**

The rate of cellular fatty acid uptake is short-term (i.e., minutes) regulated by the subcellular recycling of CD36 between endosomes and the plasma membrane. This recycling is governed by the activity of vacuolar-type H^+^-ATPase (v-ATPase) in the endosomal membrane via assembly and disassembly of two subcomplexes. The latter process is being influenced by metabolic substrates including fatty acids, glucose and specific amino acids, together resulting in a dynamic interplay to modify cellular substrate preference and uptake rates. Moreover, in cases of metabolic disease v-ATPase activity was found to be affected while interventions aimed at normalizing v-ATPase functioning had therapeutic potential.

**Summary:**

The emerging central role of CD36 in cellular lipid homeostasis and recently obtained molecular insight in the interplay among metabolic substrates indicate the applicability of CD36 as target for metabolic modulation therapy in disease. Experimental studies already have shown the feasibility of this approach.

## INTRODUCTION

Cluster of differentiation 36 (CD36) is an 88 kDa transmembrane glycoprotein that functions as a receptor mediating the binding and cellular uptake of, among others, thrombospondin, oxidized lipids and long-chain fatty acids [1,2]. CD36 is a member of a superfamily of scavenger receptor proteins class B, and officially designated as SR-B2 [3]. The expression pattern of CD36 reflects its functioning in lipid metabolism and innate immunity, specifically in intestinal fat absorption, lipid storage in adipose tissue and lipid utilization by cardiac and skeletal muscle including derangements seen in metabolic disorders such as obesity and diabetes, atherothrombotic disease and chronic kidney disease [2,4]. CD36 also is involved in neurodegenerative disorders such as Alzheimer's disease and multiple sclerosis [5^▪^,^6^].

Recent advances in our understanding of the molecular mechanism underlying CD36 functioning and regulation has pointed towards a prominent and pivotal role for CD36 in cellular fatty acid homeostasis. In this review, we will highlight these recent developments, focusing on fatty acid metabolism in the heart and changes therein in cardiac disease. 

**Box 1:**
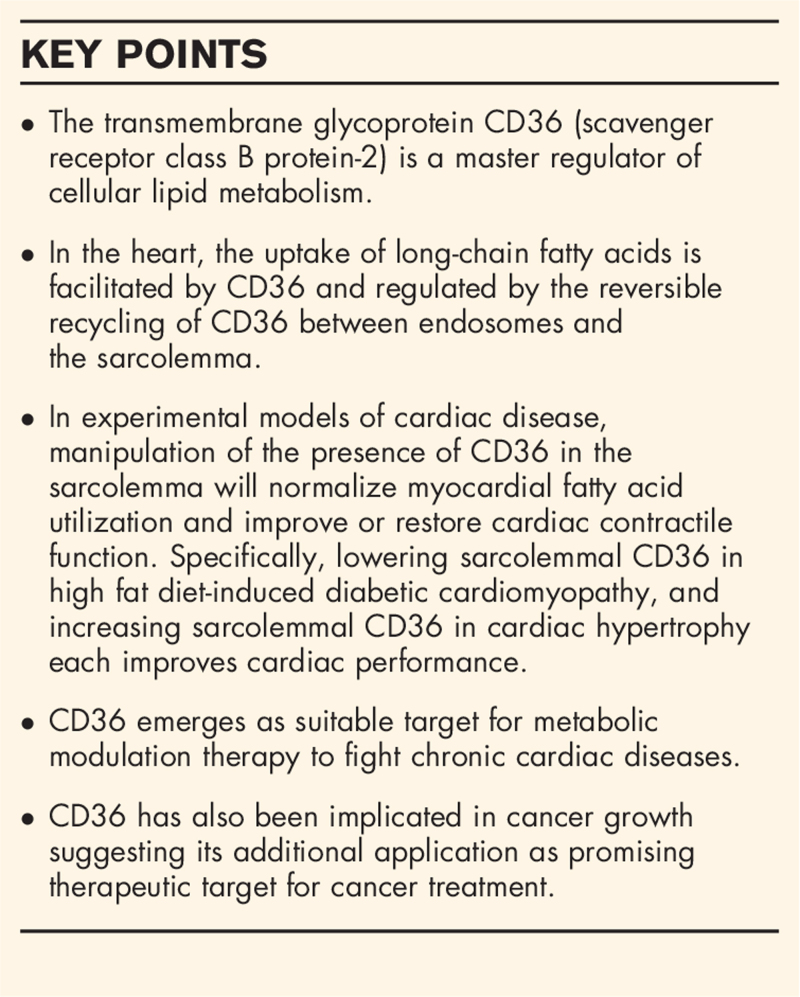
no caption available

## CD36 REGULATES CELLULAR FATTY ACID UPTAKE

**FIGURE 1:**
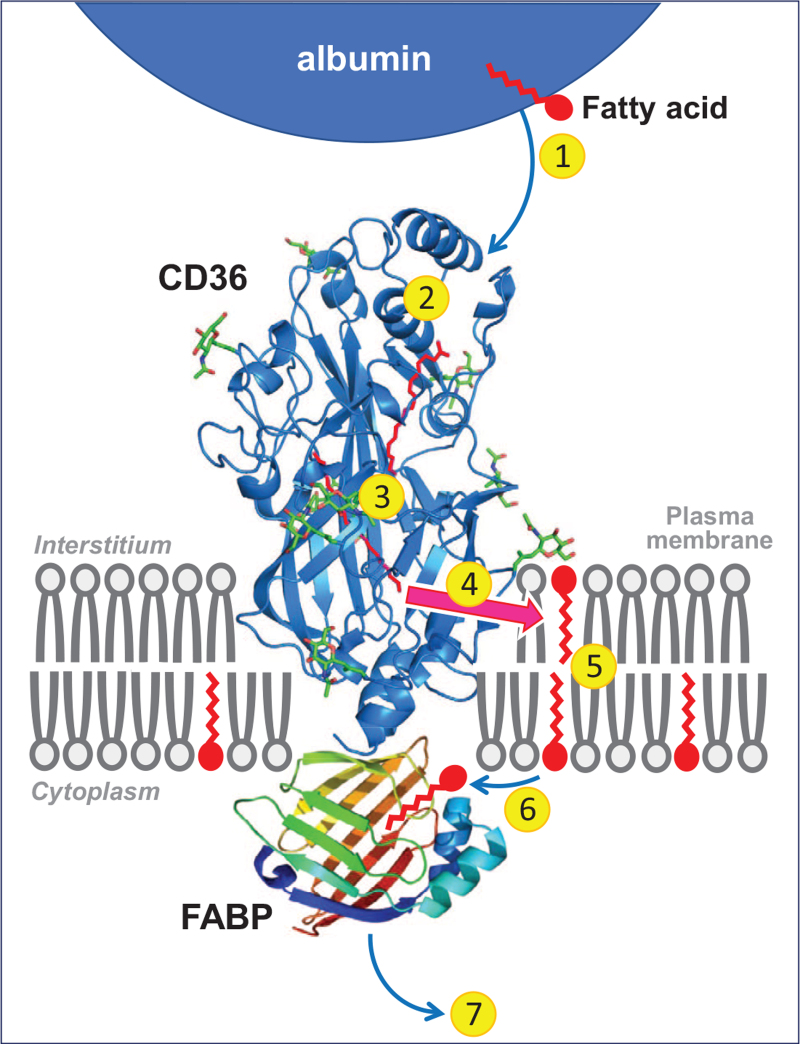
Cartoon illustrating the sequential steps involved in the uptake of long-chain fatty acids by cells. 1. Release of fatty acids from (interstitial) albumin. 2. Binding in the hydrophobic cavity of CD36 which can accommodate up to two fatty acids at a time. 3. Guidance of the fatty acid through the CD36 ectodomain interior to pass the unstirred water layer and be exposed to the plasma membrane surface. 4. Exit of the fatty acid from CD36 to the outer leaflet of the phospholipid bilayer. 5. Transmembrane translocation (‘flip-flop’) of single fatty acids. 6. Desorption of fatty acids from the inner leaflet of the phospholipid bilayer and binding to the interior of FABP_c_ that is anchored by binding to the intracellular part of CD36. 7. Diffusion into the soluble cytoplasm of the fatty acid–FABP_c_ complex towards sites of intracellular fatty acid metabolism. Note that proteins and membranes, and their putative mutual interactions are not drawn to scale. Reproduced with permission from [7^▪^]. CD36, cluster of differentiation 36. FABP_c_, cytoplasmic fatty acid-binding protein.

After intensely and for many years debating the molecular mechanism by which cells take up long-chain fatty acids, recently consensus has been reached [7^▪^]. Membrane-associated proteins such as CD36 act as an acceptor for fatty acids to promote the partitioning and their delivery to the outer leaflet of the plasma membrane. The subsequent transmembrane movement of fatty acids occurs by passive diffusion (‘flip–flop’) without the need for membrane proteins to facilitate this diffusional process. However, at the inner side of the membrane CD36 is needed to facilitate the desorption of fatty acids and their subsequent binding to cytoplasmic fatty acid-binding protein (FABP_c_). As a result, the molecular mechanism of cellular fatty acid uptake comprises an integration of simple diffusion (within the lipid bilayer of the membrane) and facilitated diffusion (capture of fatty acids into the membrane and their release from the membrane), whereby the presence of CD36 in the plasma membrane dramatically accelerates the overall rate of fatty acid uptake (or their release from adipocytes) (Fig. 1) [7^▪^]. In the plasma membrane, CD36 is localized in lipid rafts and interacts with caveolins, which may further optimize cellular fatty acid uptake [8], for instance through caveolae-dependent endocytosis of CD36 [9^▪▪^]. Nevertheless, it should be emphasized that the direction and actual rate of fatty acid transport across the plasma membrane is determined by the transmembrane gradient of fatty acids [10].

Although other membrane proteins have been identified to be involved in cellular fatty acid uptake, such as plasma membrane FABP (FABP_pm_) and fatty acid-transport protein (FATP) [11], in the heart CD36 appears to be the predominant fatty acid uptake facilitator, both for transendothelial fatty acid transport and fatty acid uptake into cardiomyocytes [12,13^▪^]. For instance, in patients with a various single nucleotide polymorphisms in the *CD36* gene, in-vivo myocardial fatty acid uptake was virtually absent [14] or markedly reduced [15], and mice with a targeted deletion of CD36 showed markedly reduced (–50% to –80%) cardiac fatty acid uptake both *in vitro*[16] and *in vivo*[17,18]. Furthermore, CD36-mediated sarcolemmal fatty acid transport is the rate-limiting kinetic step in overall myocardial fatty acid utilization with long-chain acyl-CoA synthetase (ACSL) and carnitine palmitoyl-transferase-I (CPT-I) serving merely permissive roles [12,19].

**FIGURE 2:**
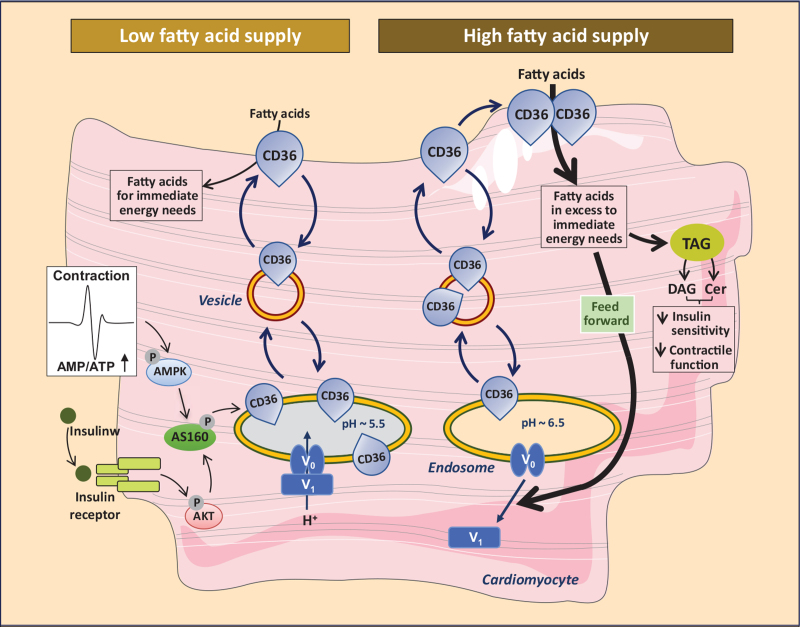
Schematic presentation of both the facilitatory and regulatory roles of CD36 in (long-chain) fatty acid uptake into cardiomyocytes. *Left part of figure*: Short-term regulation (i.e., minutes) of the rate of cellular fatty acid uptake occurs by reversible intracellular recycling (through vesicular transport) of CD36 from an endosomal storage compartment to the sarcolemma, which is triggered by changes in the frequency of muscle contraction or by plasma insulin. These latter triggers are mediated by the AMPK-activated and insulin signaling cascades, respectively, which converge at AS160. *Right part of figure*: Excess extracellular fatty acid supply triggers a series of events: increased CD36-mediated fatty acid uptake results in elevated fatty acid availability which causes the V_1_ subcomplex of v-ATPase to dissociate from the membrane-bound V_0_ subcomplex into the soluble cytoplasm. The disassembly of v-ATPase inhibits its proton pumping activity to cause an alkalinization of the endosome. Increased endosomal pH triggers the translocation of CD36 vesicles to the plasma membrane. Upon chronic fatty acid oversupply, where fatty acid uptake surpasses the metabolic needs, the increase in plasma membrane CD36 feeds forward to further increased fatty acid uptake and progressive lipid storage (TAG, triacylglyceroles). The latter is accompanied by increased levels of diacylglyceroles (DAG) and ceramides (Cer) ultimately eliciting loss of insulin sensitivity and of contractile function. CD36, cluster of differentiation 36.

Short-term regulation of the rate of cellular fatty acid uptake occurs mainly by adaptation of the presence of CD36 at the cell surface via its continuous recycling between subcellularly located endosomes and the sarcolemma. Notably, the presence of insulin or an increase in muscle contraction each stimulate, within minutes, the net translocation of CD36 from the endosomes to the sarcolemma to increase the rate of fatty acid uptake [20]. Upon removal of the trigger, CD36 is internalized immediately, and the rate of fatty acid uptake is lowered (Fig. 2). This mechanism is very similar to the regulation of myocardial glucose uptake by the recycling of glucose transporter-4 (GLUT4) between an intracellular storage depot and the sarcolemma [21]. Taken together, the uptake of the two major substrates of the heart, that is, long-chain fatty acids and glucose, is regulated in a similar manner and involves the reversible recruitment of membrane proteins CD36 and GLUT4, respectively, from intracellular stores to the sarcolemma. A proper interplay between the uptake of these two substrates is important as it has been delineated that proper cardiac contractile performance is dependent on an optimal balance between the utilization of fatty acids and that of glucose [22^▪^].

## MOLECULAR MECHANISM OF SUBCELLULAR RECYCLING OF CD36

The molecular mechanism underlying the rapid adjustment of cell surface CD36 has been elucidated and involves the activity of vacuolar-type H^+^-ATPase (v-ATPase), a large (830 kDa) multimeric protein complex in the endosomal membrane that acts as an ATP-dependent proton pump and is responsible for endosomal acidification in every mammalian cell type, including cardiomyocytes. V-ATPase consists of 14 subunits organized in two subcomplexes, that is, an integral membrane-associated subcomplex (V_0_) of six subunits forming the proton translocation channel, and a peripheral or cytoplasmic subcomplex (V_1_) of eight subunits, which contains the ATP-binding pocket and forms the ATP-driven rotor [reviewed in 23^▪▪^]. The activity of v-ATPase is controlled by various mechanisms, reflecting the diversity of its function. Instantaneous modulation of v-ATPase activity is achieved by regulation of the assembly of the V_0_ and V_1_ domains in response to nutrient availability, growth factor stimulation or cellular differentiation, whereby the V_1_ domain undergoes cycles of rapid and reversible movement away from the endosomal membrane into the soluble cytoplasm (Fig. 2) [23^▪▪^]. Considerable insight has recently emerged concerning the cellular signaling pathways controlling this regulated (re-)assembly.

Both long-chain fatty acids and glucose affect the assembly state of v-ATPase and, therefore, its proton pumping activity. For instance, palmitate exposure of isolated cardiomyocytes induces the disassembly of v-ATPase into its two subcomplexes, resulting in inhibition of v-ATPase activity and, therefore, decreased endosomal acidification [24]. This was accompanied by an increased net translocation of CD36 to the sarcolemma and consequently an increased fatty acid uptake rate, leading to a feed-forward cycle of further increased CD36 translocation and fatty acid uptake (Fig. 2) [24]. The corollary is that v-ATPase activity is required for CD36 retention in the endosomes and that stimulation of this activity will limit the rate of cellular lipid uptake, while v-ATPase inhibition will elicit an increased rate of fatty acid uptake that, when uncontrolled, may lead to excess lipid accumulation and lipid-induced abnormalities.

Interestingly, glucose acts as a stimulus for v-ATPase assembly [25] resulting in net endosomal CD36 retention and a decreased rate of fatty acid uptake [26]. The opposite regulation of v-ATPase activity by lipids and by glucose suggests that the assembly state of v-ATPase reflects the ratio between the uptake of fatty acids and glucose at any given nutritional condition [27]. Because fatty acids and glucose are the principal substrates for myocardial energy provision, together covering up to 90% of ATP production [28], the ability of v-ATPase to sense (alterations in) both intracellular fatty acid and glucose concentrations makes v-ATPase a central regulator of myocardial substrate preference and energy substrate metabolism.

Recently, it was observed that specific amino acids also stimulate v-ATPase (re-)assembly to retain CD36 in the endosomes and reduce myocardial fatty acid uptake [29^▪^]. These novel findings combined indicate the application of specific amino acid supplementation for tuning of the contributions of fatty acids and of glucose to their current cellular demand so as to avoid both fatty acid overload (lipotoxicity) and glucose overload (glucotoxicity).

## CD36 IN LIPID SIGNALING

Long-chain fatty acids are not only energy substrates but also have potent effects on cellular signal transduction and gene programming. This provides CD36 as main cardiac fatty acid transporter also with an important role in lipid signaling. Long-chain fatty acids are ligands for nuclear receptors such as peroxisome proliferator-activated receptor (PPAR), which are known to stimulate the expression of proteins involved in cellular lipid metabolism [30,31]. Interestingly, in this way CD36 facilitates the regulation of its own expression. CD36 signaling has been observed also to regulate the activity of AMP-activated kinase (AMPK) to coordinate skeletal muscle fatty acid uptake with its subsequent mitochondrial oxidation [32]. Reciprocally, AMPK was reported to increase intestinal fatty acid uptake by upregulating CD36 protein expression and stimulating its translocation to the membrane simultaneously [33^▪^]. These findings indicate a dynamic interplay between CD36 and AMPK. Finally, CD36 has been reported to interact with the insulin receptor to promote tyrosine phosphorylation of this receptor and enhance downstream insulin signaling, a process that is suppressed by fatty acids [32]. These insights suggest a role for CD36 in optimal insulin responsiveness, and by inference in the regulation of cardiac and muscular glucose utilization.

## CD36 IN THE PATHOGENESIS OF (CARDIO)METABOLIC DISEASE

Disturbances in myocardial metabolism are increasingly being recognized as crucial drivers of the development and progression of heart disease [34,35]. Furthermore, interventions directed at normalizing metabolism are emerging as promising and effective therapeutic options in the treatment of heart failure [36]. Given the key regulatory role of CD36 in lipid metabolism, it logically follows that CD36 is involved in the development of disease and may be a suitable target for so-called metabolic modulation therapy. Indeed, in recent years various studies have documented such role for CD36 in metabolic disease development.

Detailed investigations by several independent research groups have revealed that CD36 plays a key early role in the development of obesity-induced insulin resistance and type 2 diabetes. In the heart, this pathological condition eventually elicits contractile dysfunction and, therefore, is designated as diabetic cardiomyopathy [37]. During consumption of a Western (high fat-containing) diet and in obesity the heart is subject to a chronic oversupply of fatty acids, which triggers the enhanced recruitment of CD36 to the sarcolemma. By the feed-forward principle explained above, this leads to a net relocation of CD36 from endosomes to the sarcolemma, and an almost complete shift in myocardial substrate preference towards long-chain fatty acids at the expense of glucose [38–40,41^▪▪^]. After starting a high-fat diet, the subcellular redistribution of CD36 and the concomitant increase in fatty acid uptake rate occur very rapidly, that is, within 3 days, before any other change in metabolism [42]. The excess incoming fatty acids exceeds the capacity for mitochondrial oxidation and then will be stored into triacylglycerols and converted into lipid metabolites (ceramides, diacylglycerol) known to inhibit insulin signaling, thus causing insulin resistance [24,41^▪▪^]. Thereafter, recruitment of CD36 and glucose transporter GLUT4 to the sarcolemma become impaired (insulin resistance) (Fig. 2). Yet, given that CD36 is already at the sarcolemma, this will selectively result in a markedly lower rate of glucose uptake [42]. Taken together, in the heart towards development of insulin resistance, increases in CD36-mediated fatty acid uptake precede and are causal to decreased rates of GLUT4-mediated glucose uptake, and culminalte into a juxtaposed subcellular localization of CD36 and GLUT4 in cardiomyocytes (with CD36 being mostly at the cell surface and GLUT4 mainly imprisoned intracellularly) as a further step towards overt diabetic cardiomyopathy. The heart then relies almost completely on fatty acids for metabolic energy provision, cannot take up sufficient amounts of glucose, and develops contractile dysfunction [43^▪^]. The pivotal early role of CD36 in this cascade of events is evidenced by the observations that genetic ablation of CD36 [44,45] or blocking its activity by specific inhibitors (e.g., sulfo-*N*-succinimidyl-oleate) [46–48] or antibodies [49] fully prevent the loss of contractile function of cardiomyocytes.

Conversely, stimulation of CD36 transport activity may play a role in the development of diseases that are elicited by a decreased and subnormal lipid metabolism, such as seen during the development of cardiac hypertrophy and failure when the heart switches to excess utilization of glucose at the expense of fatty acids [50]. In this condition, a high fat diet-induced increase in CD36-mediated fatty acid uptake will re-balance myocardial fatty acid and glucose utilization, and restore cardiac contractile function [51].

## CD36 AS TARGET FOR METABOLIC INTERVENTION

Given the early pivotal role of CD36 in metabolic disease development, in particular when related to altered lipid metabolism, several investigators have started to apply CD36 as a target for metabolic intervention therapy in order to normalize cardiac metabolism and restore contractile function [52]. As described above, specifically inhibiting sarcolemmal CD36 is effective to lower cellular fatty acid uptake, but is not regarded a suitable systemic approach as such inhibition would also impair fatty acid uptake in other organs, especially adipose tissue, and may affect other known functions of CD36, such as scavenging of oxidized low-density lipoprotein by macrophages [1]. Instead, a preferable approach is to manipulate the subcellular recycling of CD36 thereby modulating its presence at the sarcolemma and to do so in a tissue-specific manner [52].

The recent disclosure of v-ATPase as regulator of CD36 recycling makes this endosomal enzyme an interesting candidate for modulation of sarcolemmal CD36 content. Indeed, as outlined above, specific amino acids increase myocardial v-ATPase activity thereby reducing sarcolemmal CD36, and ultimately preventing or restoring lipid overexposure-induced contractile dysfunction [29^▪^]. However, whether these amino acids act specifically on the heart or would also affect v-ATPase in other tissues has not yet been addressed. On the other hand, the 14 subunits of v-ATPase show marked tissue-specific differences, suggesting that designing cell- or tissue-specific drugs to alter its activity is feasible [53^▪^].

It has been estimated that some 50−60 distinct proteins are involved in subcellular vesicular trafficking pathways, such as coat proteins, adaptor proteins, and motor proteins that enable transport of vesicles along the filamentous network from one specific address to the other [27,54]. The CD36-dedicated vesicular trafficking machinery has only partly been elucidated, but already revealed that isoforms of vesicle-associated membrane proteins (VAMPs) bring specificity to myocardial CD36 recycling [27,55]. Thus, VAMP isoform 4 (VAMP4) was found to specifically participate in myocardial CD36 traffic, and not in GLUT4 traffic, suggesting the possibility of using VAMP4 as target to manipulate CD36-mediated fatty acid uptake without affecting GLUT4 translocation or glucose uptake [55]. Genetic ablation of VAMP4 in cardiomyocytes in suspension markedly decreased the rate of fatty acid uptake without affecting glucose uptake, which provides proof-of-concept for this approach [55]. As a result, pharmacologically inhibiting VAMP4 is expected to be an effective approach to lower CD36 translocation to the sarcolemma with the aim to counteract myocellular lipid accumulation and associated cardiac contractile dysfunction.

## CONCLUDING REMARKS AN FUTURE PERSPECTIVES

**FIGURE 3:**
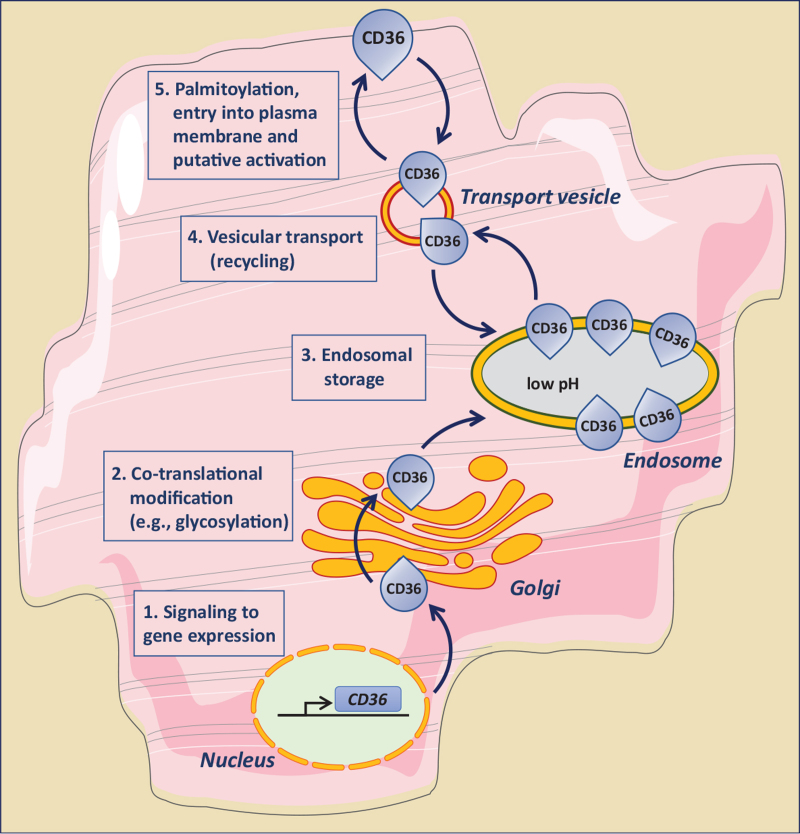
Schematic presentation of the five distinct levels at which the functioning of CD36 in cellular fatty acid uptake can be modulated. 1. Intervention of signaling pathways affecting CD36 gene expression. 2. Intervention in co-translational modification of CD36, in particular its glycosylation. 3. Manipulation of the endosomal storage of CD36. 4. Modulation of the reversible vesicular transport of CD36 between an endosomal storage compartment and the plasma membrane (subcellular recycling). 5. Influencing CD36 palmitoylation, its insertion into the membrane bilayer, and putative activation (e.g., interaction with other membrane-associated proteins). CD36, cluster of differentiation 36.

At present there is conclusive evidence that the transmembrane glycoprotein CD36 fulfills a pivotal facilatory and regulatory role in cellular lipid metabolism, both in health and in the development of metabolic disease. Although there is ongoing progress in understanding the underlying molecular mechanisms of CD36 functioning, still much needs to be learned especially because its regulation occurs at multiple levels, that is, gene expression, posttranslational modification, storage in endosomes, intracellular vesicular transport, entry into the plasma membrane and putative activation (Fig. 3). As a result, modulation of its content at the plasma membrane to alter lipid metabolism effectively so as to prevent or restore lipid-induced cellular malfunction (e.g., lipid-induced myocardial contractile dysfunction), awaits the further exploration of several aspects of CD36 action before its routine application as effective target for metabolic modulation therapy. These aspects include the trafficking machinery involved in the translocation of CD36 from endosomes to the plasma membrane and *vice versa*, the putative activation of CD36 after its arrival at the plasma membrane and required for optimal function [56], the presumable interaction with membrane lipids and with other membrane proteins (such as caveolin and FABP_pm_) [57,58], and the significance of posttranslational modification of CD36 for its functioning [59,60^▪^]. With respect to the latter, CD36 requires palmitoylation for its proper insertion into the membrane but its hyper-palmitoylation may occur during lipid oversupply and then will contribute to lipid accumulation and the development of insulin resistance [9^▪▪^,^61^]. Such additional insights, together with the availability of the structure and membrane topology of CD36 [62] will accelerate the design of small-molecule compounds to be used as pharmacological agents to specifically influence CD36 functioning [63^▪^].

In this review, we focused on the role of CD36 in the heart. Importantly, however, CD36 has also been reported to be involved in cancer growth [64–67] while targeting lipid metabolism of cancer cells appears a promising therapeutic strategy for cancer treatment [68,69,70^▪^]. Most notably, it was found that CD36 marks a subpopulation of cancer cells with unique metastasis-initiating potential, highlighting a key role of lipid metabolism in metastatic colonization [71]. This has led to the development of antitumor drugs targeting the ligand receptor function of CD36, which failed in clinical trials mostly because of adverse events [reviewed in 69], further indicating the need for detailed insight in CD36 regulation and function in specific tissues before its routine application as therapeutic target in the clinic.

## Acknowledgements

*None.*

### Financial support and sponsorship

*Miranda Nabben is the recipient of a Dekker Grant from the Dutch Heart Foundation (nr. 2019T041).*

### Conflicts of interest

*There are no conflicts of interest.*

## REFERENCES AND RECOMMENDED READING

Papers of particular interest, published within the annual period of review, have been highlighted as:

▪ of special interest
▪▪ of outstanding interest

## References

[1] Collot-Teixeira S Martin J McDermott-Roe C. CD36 and macrophages in atherosclerosis (review). Cardiovasc Res 2007; 75:468–477. 17442283 10.1016/j.cardiores.2007.03.010

[2] Silverstein RL Febbraio M. CD36, a scavenger receptor involved in immunity, metabolism, angiogenesis, and behavior. Sci Signal 2009; 2:re3. 19471024 10.1126/scisignal.272re3 PMC2811062

[3] Prabhudas M Bowdish D Drickamer K. Standardizing scavenger receptor nomenclature. J Immunol 2014; 192:1997–2006. 24563502 10.4049/jimmunol.1490003 PMC4238968

[4] Yang X Okamura D Lu X. CD36 in chronic kidney disease: novel insights and therapeutic opportunities. Nat Rev Nephrol 2017; 13:769–781. 28919632 10.1038/nrneph.2017.126

[5▪] Dobri A-M Dudau M Enciu A-M Inescu ME. CD36 in Alzheimer's disease: an overview of molecular mechanisms and therapeutic targeting. Neuroscience 2021; 453:301–311. 33212223 10.1016/j.neuroscience.2020.11.003

[6] Grajchen E Wouters E Van de Haterd B. CD36-mediated uptake of myelin debris by macrophages and microglia reduces neuroinflammation. J Neuroinflamm 2020; 17:224. 10.1186/s12974-020-01899-x PMC7384221 32718316

[7▪] Glatz JFC Luiken JJFP. Time for a détente in the war on the mechanism of cellular fatty acid uptake. J Lipid Res 2020; 61:1300–1303. 32873748 10.1194/jlr.6192020LTE PMC7469886

[8] Su X Abumrad NA. Cellular fatty acid uptake: a pathway under construction. Trends Endocrinol Metab 2009; 20:72–77. 19185504 10.1016/j.tem.2008.11.001 PMC2845711

[9▪▪] Hao J-W Wang J Guo H. CD36 facilitates fatty acid uptake by dynamic palmitoylation-regulated endocytosis. Nat Commun 2020; 11:4765. 32958780 10.1038/s41467-020-18565-8 PMC7505845

[10] Luiken JJFP Van Nieuwenhoven FA America G. Uptake and metabolism of palmitate by isolated cardiac myocytes from adult rats: involvement of sarcolemmal proteins. J Lipid Res 1997; 38:745–758. 9144089

[11] Mallick R Basak S Dutta-Roy AK. Fatty acids and evolving roles of their proteins in neurological, cardiovascular disorders and cancers. Progr Lipid Res 2021; 83:101116. 10.1016/j.plipres.2021.101116 34293403

[12] Glatz JFC Luiken JJFP Bonen A. Membrane fatty acid transporters as regulators of lipid metabolism: Implications for metabolic disease. Physiol Rev 2010; 90:367–417. 20086080 10.1152/physrev.00003.2009

[13▪] Abumrad NA Cabodevilla AG Samovski D. Endothelial cell receptors in tissue lipid uptake and metabolism. Circ Res 2021; 128:433–450. 33539224 10.1161/CIRCRESAHA.120.318003 PMC7959116

[14] Tanaka T Nakata T Oka T. Defect in human myocardial long-chain fatty acid uptake is caused by FAT/CD36 mutations. J Lipid Res 2001; 42:751–759. 11352982

[15] Hames KC Vella A Kemp BJ Jensen MD. Free fatty acid uptake in humans with CD36 deficiency. Diabetes 2014; 63:3606–3614. 24917573 10.2337/db14-0369 PMC4207394

[16] Habets DD Coumans WA Voshol PJ. AMPK-mediated increase in myocardial long-chain fatty acid uptake critically depends on sarcolemmal CD36. Biochem Biophys Res Commun 2007; 355:204–210. 17292863 10.1016/j.bbrc.2007.01.141

[17] Abumrad NA Goldberg IJ. CD36 actions in the heart: lipids, calcium, inflammation, repair and more? Biochim Biophys Acta 2016; 1861:1442–1449. 27004753 10.1016/j.bbalip.2016.03.015 PMC4983248

[18] Coburn CT Knapp FF Febbraio M. Defective uptake and utilization of long chain fatty acids in muscle and adipose tissues of CD36 knockout mice. J Biol Chem 2000; 275:32523–32529. 10913136 10.1074/jbc.M003826200

[19] Luiken JJFP Niessen HE Coort SLM. Etomoxir-induced partial carnitine palmitoyltransferase-I (CPT-I) inhibition in vivo does not alter cardiac long-chain fatty acid uptake and oxidation rates. Biochem J 2009; 419:447–455. 19138173 10.1042/BJ20082159

[20] Bonen A Luiken JJFP Arumugam Y. Acute regulation of fatty acid uptake involves the cellular redistribution of fatty acid translocase. J Biol Chem 2000; 275:14501–14508. 10799533 10.1074/jbc.275.19.14501

[21] Klip A McGraw TE James DE. Thirthy sweet years of GLUT4. J Biol Chem 2019; 294:11369–11381. 31175156 10.1074/jbc.REV119.008351 PMC6663870

[22▪] Glatz JFC Nabben M Young ME. Re-balancing cellular energy substrate metabolism to mend the failing heart. Biochim Biohys Acta 2020; 1866:165579. 10.1016/j.bbadis.2019.165579 PMC7586321 31678200

[23▪▪] Eaton AF Merkulova M Brown D. The H^+^-ATPase (V-ATPase): from proton pump to signaling complex in health and disease. Am J Physiol Cell Physiol 2021; 320:C392–C414. 33326313 10.1152/ajpcell.00442.2020 PMC8294626

[24] Liu Y Steinbusch LKM Nabben N. Palmitate-induced vacuolar-type H^+^-ATPase inhibition feeds forward into insulin resistance and contractile dysfunction. Diabetes 2017; 66:1521–1534. 28302654 10.2337/db16-0727

[25] McGuire C Stransky L Cotter K Forgac M. Regulation of v-ATPase activity. Front Biosci 2017; 22:609–622. 10.2741/4506 27814636

[26] Wang S Wong L-Y Neumann D. Augmenting vacuolar H^+^-ATPase function prevents cardiomyocytes from lipid-overload induced dysfunction. Int J Mol Sci 2020; 21:1520. 10.3390/ijms21041520 PMC7073192 32102213

[27] Luiken JJFP Nabben M Neumann D Glatz JFC. Understanding the distinct subcellular trafficking of CD36 and GLUT4 during the development of myocardial insulin resistance. Biochim Biophys Acta 2020; 1866:165775. 10.1016/j.bbadis.2020.165775 32209364

[28] Stanley WC Recchia FA Lopaschuk GD. Myocardial substrate metabolism in the normal and failing heart. Physiol Rev 2005; 85:1093–1129. 15987803 10.1152/physrev.00006.2004

[29▪] Wang S Schianchi F Neumann. Specific amino acid supplementation rescues the heart from lipid overload-induced insulin resistence and contractile dysfunction by targeting the endosomal mTOR–v-ATPase axis. Mol Metab 2021; 53:101293. 34265467 10.1016/j.molmet.2021.101293 PMC8350375

[30] Neels GJ Grimaldi PA. Physiological functions of peroxisome proliferator-activated receptor β. Physiol Rev 2014; 94:795–858. 24987006 10.1152/physrev.00027.2013

[31] Montaigne D Butruille L Staels B. PPAR control of metabolism and cardiovascular functions. Nat Rev Cardiol 2021; 18:809–823. 34127848 10.1038/s41569-021-00569-6

[32] Samovski D Dhule P Pietka T. Regulation of insulin receptor pathway and glucose metabolism by CD36 signaling. Diabetes 2018; 67:1272–1284. 29748289 10.2337/db17-1226 PMC6014550

[33▪] Wu W Wang S Liu Q. AMPK facilitates intestinal long-chain fatty acid uptake by manipulating CD36 expression and translocation. FASEB J 2020; 34:4852–4869. 32048347 10.1096/fj.201901994R

[34] Neubauer S. The failing heart – an engine out of fuel. N Engl J Med 2007; 356:1140–1151. 17360992 10.1056/NEJMra063052

[35] Taegtmeyer H Young ME Lopaschuk GD. Assessing cardiac metabolism: a scientific statement from the American Heart Association. Circ Res 2016; 118:1659–1701. 27012580 10.1161/RES.0000000000000097 PMC5130157

[36] Rosano GMC Vitale C. Metabolic modulation of cardiac metabolism in heart failure. Cardiac Fail Rev 2018; 4:99–103. 10.15420/cfr.2018.18.2 PMC6125709 30206484

[37] Boudina S Abel ED. Diabetic cardiomyopathy, causes and effects. Rev Endocr Metab Disord 2010; 11:31–39. 20180026 10.1007/s11154-010-9131-7 PMC2914514

[38] Ouwens DM Diamant M Fodor M. Cardiac contractile dysfunction in insulin-resistant rats fed a high fat diet is associated with elevated CD36-mediated fatty acid uptake and esterification. Diabetologia 2007; 50:1938–1948. 17639306 10.1007/s00125-007-0735-8 PMC2039861

[39] Aguer C Mercier J Yong Wai Man C. Intramyocellular lipid accumulation is associated with permanent relocation *ex vivo* and *in vitro* of fatty acid translocase (FAT)/CD36 in obese patients. Diabetologia 2010; 53:115–1163. 20333349 10.1007/s00125-010-1708-x

[40] Aguer C Foretz M Lantier L. Increased FAT/CD36 cycling and lipid accumulation in myotubes derived from obese type 2 diabetic patients. PLoS One 2011; 6:e28981. 22194967 10.1371/journal.pone.0028981 PMC3241688

[41▪▪] Zhu B Li M-Y Lin Q. Lipid oversupply induces CD36 sarcolemmal translocation via dual modulation of PKCζ and TBC1D1: an early event prior to insulin resistance. Theranostics 2020; 10:1332–1354. 31938068 10.7150/thno.40021 PMC6956797

[42] Bonen A Jain SS Snook LA. Extremely rapid increase in fatty acid transport and intramyocellular lipid accumulation but markedly delayed insulin resistance after high fat feeding in rats. Diabetologia 2015; 58:2381–2391. 26197708 10.1007/s00125-015-3691-8

[43▪] Zhang X Fan J Li H. CD36 signaling in diabetic cardiomyopathy. Aging Dis 2021; 12:826–840. 34094645 10.14336/AD.2020.1217 PMC8139204

[44] Steinbusch LKM Luiken JJFP Vlasblom R. Absence of fatty acid transporter CD36 protects against Western-type diet-related cardiac dysfunction following pressure overload in mice. Am J Physiol Endocrinol Metab 2011; 301:E618–E628. 21712535 10.1152/ajpendo.00106.2011

[45] Sung MM Koonen DP Soltys CL. Increased CD36 expression in middle-aged mice contributes to obesity-related cardiac hypertrophy in the absence of cardiac dysfunction. J Mol Med 2011; 89:459–469. 21387178 10.1007/s00109-010-0720-4

[46] Coort SLM Willems J Coumans WA. Sulfo-*N*-succinimidyl esters of long chain fatty acids specifically inhibit fatty acid translocase (FAT/CD36)-mediated cellular fatty acid uptake. Mol Cell Biochem 2002; 239:213–219. 12479588

[47] Kuda O Pietka TA Demianova Z. Sulfo-*N*-succinimidyl oleate (SSO) inhibits fatty acid uptake and signaling for intracellular calcium via binding CD36 lysine 164. J Biol Chem 2013; 288:15547–15555. 23603908 10.1074/jbc.M113.473298 PMC3668716

[48] Yang J Park KW Cho S. Inhibition of the CD36 receptor reduces visceral fat accumulation and improves insulin resistance in obese mice carrying the BDNF-*Val66Met* variant. J Biol Chem 2018; 293:13338–13348. 29914985 10.1074/jbc.RA118.002405 PMC6109932

[49] Angin Y Steinbusch LKM Simons PJ. CD36 inhibition prevents lipid accumulation and contractile dysfunction in rat cardiomyocytes. Biochem J 2012; 448:43–53. 22780108 10.1042/BJ20120060

[50] Geraets IME Glatz JFC Luiken JJFP Nabben M. Pivotal role of membrane substrate transporters on the metabolic alterations in the pressure-overlaoded heart. Cardiovasc Res 2019; 115:1000–1012. 30938418 10.1093/cvr/cvz060

[51] Dirkx E Van Eys GJJM Schwenk RW. Protein kinase-D1 overexpression prevents lipid-induced cardiac insulin resistance. J Mol Cell Cardiol 2014; 76:208–217. 25173922 10.1016/j.yjmcc.2014.08.017

[52] Glatz JFC Luiken JJFP Nabben M. CD36 (SR-B2) as a target to treat lipid overload-induced cardiac dysfunction. J Lipid Atheroscler 2020; 9:66–78. 32821722 10.12997/jla.2020.9.1.66 PMC7379071

[53▪] Santos-Pereira C Rodrigues LR Corte-Real M. Emerging insights on the role of V-ATPase in human diseases: therapeutic challenges and opportunities. Med Res Rev 2021; 41:1927–1964. 33483985 10.1002/med.21782

[54] Chi RJ Harrison MS Burd CG. Biogenesis of endosome-derived transport carriers. Cell Mol Life Sci 2015; 72:3441–3455. 26022064 10.1007/s00018-015-1935-x PMC4890637

[55] Schwenk RW Dirkx E Coumans WA. Requirement for distinct vesicle-associated membrane proteins in insulin- and AMP-activated protein kinase (AMPK)-induced translocation of GLUT4 and CD36 in cultured cardiomyocytes. Diabetologia 2010; 53:2209–2219. 20582536 10.1007/s00125-010-1832-7 PMC2931635

[56] Angin Y Schwenk RW Nergiz-Unal R. Calcium signaling recruits substrate transporters GLUT4 and CD36 to the sarcolemma without increasing cardiac substrate uptake. Am J Physiol Endocrinol Metab 2014; 307:E225–E236. 24895286 10.1152/ajpendo.00655.2013

[57] Ring A Le Lay S Pohl J. Caveolin-1 is required for fatty acid translocase (FAT/CD36) localization and function at the plasma membrane of mouse embryonic fibroblasts. Biochim Biophys Acta 2006; 1761:416–423. 16702023 10.1016/j.bbalip.2006.03.016

[58] Glatz JFC Luiken JJFP. Dynamic role of the transmembrane glycoprotein CD36 (SR-B2) in cellular fatty acid uptake and utilization. J Lipid Res 2018; 59:1084–1093. 29627764 10.1194/jlr.R082933 PMC6027920

[59] Luiken JJFP Chanda D Nabben M. Posttranslational modifications of CD36 (SR-B2): Implications for regulation of myocellular fatty acids uptake. Biochim Biophys Acta 2016; 1862:2253–2258. 27615427 10.1016/j.bbadis.2016.09.004

[60▪] Shu H Peng Y Hang W. The role of CD36 in cardiovascular disease. Cardiovasc Res 2022; 118:115–129. 33210138 10.1093/cvr/cvaa319 PMC8752351

[61] Schianchi F Glatz JFC Navarro Gascon A. Putative role of protein palmitoylation in cardiac lipid-induced insulin resistance. Int J Mol Sci 2020; 21:9438. 10.3390/ijms21249438 PMC7764417 33322406

[62] Hsieh FL Turner L Bolla JR. The structural basis for CD36 binding by the malaria parasite. Nat Commun 2016; 7:12837. 27667267 10.1038/ncomms12837 PMC5052687

[63▪] Glatz JFC Wang F Nabben M Luiken JJFP. CD36 as a target for metabolic modulation therapy in cardiac disease. Expert Opin Ther Targets 2021; 25:393–400. 34128755 10.1080/14728222.2021.1941865

[64] Hale JS Otvos B Sinyuk M. Cancer stem cell-specific scavenger receptor CD36 drives glioblastoma progression. Stem Cells 2014; 32:1746–1758. 24737733 10.1002/stem.1716 PMC4063873

[65] Zhao J Zhi Z Wang C. Exogenous lipids promote the growth of breast cancer cells via CD36. Oncol Rep 2017; 38:2105–2115. 28765876 10.3892/or.2017.5864 PMC5652970

[66] Pan J Fan Z Wang Z. CD36 mediates palmitate acid-induced metastasis of gastric cancer via AKT/GSK-3β/β-catenin pathway. J Exp Clin Cancer Res 2019; 38:52. 30717785 10.1186/s13046-019-1049-7 PMC6360779

[67] Tanase C Gheorghisan-Galateanu A-A Popescu ID. CD36 and CD97 in pancreatic cancer versus other malignancies. Int J Mol Sci 2020; 21:5656. 10.3390/ijms21165656 PMC7460590 32781778

[68] Liu Q Luo Q Halim A Song G. Targeting lipid metabolism of cancer cells: a promising therapeutic strategy for cancer. Cancer Lett 2017; 401:39–45. 28527945 10.1016/j.canlet.2017.05.002

[69] Wang J Li Y. CD36 tango in cancer: signaling pathways and functions. Theranostics 2019; 9:4893–4908. 31410189 10.7150/thno.36037 PMC6691380

[70▪] Munir R Lisec J Swinnen JV Zaidi N. Too complex to fail? Targeting fatty acid metabolism for cancer therapy. Progr Lipid Res 2022; 85:101143. 10.1016/j.plipres.2021.101143 34856213

[71] Pascual G Avgustinova A Mejetta S. Targeting metastasis-initiating cells through the fatty acid receptor CD36. Nature 2017; 541:41–45. 27974793 10.1038/nature20791

